# *Enterococcus faecalis* bacteraemia and infective endocarditis - what are we missing?

**DOI:** 10.1016/j.clinpr.2023.100336

**Published:** 2024-01

**Authors:** Clemency Nye, Alice Maxwell, Harriet Hughes, Jonathan Underwood

**Affiliations:** a Public Health Wales, University Hospital of Wales, Heath Park, Cardiff, CF14 XW; b Cardiff and Vale University Health Board, University Hospital of Wales, Heath Park, Cardiff, CF14 XW; c Division of Infection and Immunity, Cardiff University

**Keywords:** *Enterococcus faecalis*, Infective endocarditis, Echocardiography

## Abstract

**Introduction:**

*Enterococcus faecalis* is an increasingly common cause of infective endocarditis, with a recent study by Dahl *et al* demonstrating a prevalence of 26% of IE when transoesophageal echo was routinely undertaken. Another study undertaken by Østergaard *et al* found that 16.7% of patients with *E. faecalis* bacteraemia developed endocarditis. Based on these findings we examined the rates of IE diagnosed in our own health board to determine if our current practice is potentially missing cases of IE and if we could improve our management of these bacteraemias.

**Methods:**

All blood cultures in patients over 18 which were positive for *E. faecalis* from October 2017 to March 2022 were reviewed. We analysed the patient characteristics, clinical outcomes and included a follow up period of 6 months to assess for recrudescence and treatment failure.

**Results:**

The rate of patients with *E. faecalis* bacteraemia diagnosed with IE was 7.1%. If polymicrobial blood cultures were excluded this rose to 13.0%. Community acquisition, patient cardiac or immune risk factors, monomicrobial culture and multiple positive blood cultures all were associated with IE. 62.1% of patients with *E. faecalis* bacteraemia did not have an echocardiogram during their admission, due to a variety of reasons.

**Discussion:**

The lower reported rate of IE in our cohort may be explained by higher proportion of CVC related infections. However, given the low rates of echocardiography and poor correlation of echocardiography use with IE risk factors, it is likely that cases of IE are being missed, particularly in those with multiple risk factors. Despite this, there was no difference in one-year survival between those diagnosed with IE vs without IE. We have delivered education sessions and introduced a multidisciplinary team meeting to discuss infective endocarditis cases to address these issues.

## Introduction

Enterococci, known previously as group D Streptococci based on their ability to group D on Lancefield grouping, are commensals of the gastrointestinal tract. Enterococcal endocarditis, of which *Enterococcus faecalis* represents 90 %, is the third most common cause of infective endocarditis (IE), after *Staphylococcus aureus* and streptococci (Murdoch, 2009). The number of relevant studies reviewing enterococcal endocarditis is small, and previous incidence rates for endocarditis in E. *faecalis* bacteraemia have varied from 3 % (Anderson, 2004) to 14 % (Pinholt, 2014). Enterococcal endocarditis is more common in the elderly and those with prosthetic valves, and is more frequently acquired nosocomially than other causes of IE (Chirouze, 2013; Fernández-Guerrero, 2002). Some studies also indicate that the rates of enterococcal endocarditis are increasing over time (Olmos, 2017).

The understanding of IE risk in *E. faecalis* bacteraemia is a rapidly evolving area with two recent studies from Denmark demonstrating significantly higher rates of IE than previously published. Østergaard in 2019 (Østergaard, 2019) conducted a retrospective review of the endocarditis incidence in patients with a bacteraemia with a *Staphylococcus, Streptococcus* or *Enterococcus* species; 4700 patients with *E. faecalis* BSI were included, and 16.7 % of patients developed IE (Østergaard, 2019). Dahl 2019 (Dahl, 2019) offered *trans*-transthoracic (TTE) or *trans*-oesophageal echocardiography (TOE) to all patients with *E. faecalis* BSI. Of 344 patients, 82 % underwent echocardiogram, with an endocarditis rate of 26 %. TTE was negative in 47 % of cases who had later confirmed endocarditis on TOE.

These findings suggest that endocarditis is being under-diagnosed in patients with *E. faecalis* bacteraemia. We have conducted a review of patients with *E. faecalis* bacteraemia to determine local practice, IE incidence and identify potential under-diagnosis.

## Methods

This was a retrospective cohort study conducted at Cardiff and Vale University Health Board, which comprises two tertiary referral centres. Blood cultures positive for *E. faecalis* from October 2017 to March 2022 were identified using a Public Health Wales microbiological database. Polymicrobial cultures with organisms additional to *E. faecalis* were included. Patients under 18 were excluded, as were patients deceased before review of the bacteraemia by a clinical microbiologist.

Both the electronic clinical infection notes and the microbiological database are all Wales systems. Although we identified our initial bacteraemias from patients with a blood culture taken in Cardiff, importantly, patients would have been identified if they re-presented to a different hospital within Wales with either a recurrent bacteraemia or endocarditis. We would not have been able to identify patients if they represented outside Wales.

Patients were followed up for at least 6 months after the bacteraemia. Recurrent bacteraemia was defined as a further blood culture(s) with *E. faecalis* from 2 weeks – 6 months after the index culture. Hospital-acquired infection was defined as a positive blood culture taken ≥ 48 h from admission. Electronic health records and the laboratory management system were used to obtain clinical information. Source of bacteraemia was defined by the study team by reviewing the microbiology clinical notes, microbiology results and, where available, discharge summaries from treating team. The death certificates of patients who died were not available. Patient risk factors for endocarditis were defined as previous endocarditis, prosthetic heart valve, abnormal native heart valves, cardiac device or immune suppression (use of steroids, chemotherapy or anti-rheumatic drug affecting the immune system) (Dahl, 2019).

Proportions were compared using the Chi-squared and Fisher’s exact tests, and continuous data was assessed using parametric and non-parametric tests as appropriate. Survival analysis and Kaplan-Meier plots were calculated using the Survminer package (Kassambara et al., 2021) and *R* v4.2.0 (R Core Team, 2022). Groups were compared using the log-rank test. P-values < 0.05 were considered statistically significant.

This study was a retrospective service evaluation of routinely collected clinical data. As such, formal Research Ethics Committee review was not required, as confirmed by the NHS Health Research Authority. Information governance approval was obtained from Public Health Wales.

## Results

Characteristics of patient group with *E. faecalis* bacteraemia.

We identified 140 patients with *E. faecalis* bacteraemia (median age 67 years). There was a male predominance, with 69.3 % being male (see table one for baseline characteritiscs).

60 % of BSIs were hospital acquired. The source of the bacteraemia was ‘unknown’ in a large proportion at 36 %, which has previously been associated with increased IE risk (Pinholt, 2014; Dahl, 2016). The most common known source was urinary (20.0 %), followed by venous catheter infection (19.3 %). Only 17.9 % of patients with a known source had microbiological confirmation with *E. faecalis* cultured from the site. Patients from haematology and renal specialties are over-represented compared to their proportion of the hospital population, at 11 % and 7 % of the total respectively.

Use of echocardiography.

37.9 % of patients with *E. faecalis* bacteraemia underwent echocardiography during admission. The median time from bacteraemia to echocardiography was 6 days. Only 4 TOEs were performed, 3 of which (75 %) demonstrated endocarditis.

Many patients with risk factors for endocarditis did not undergo echocardiography – this included 52 % of patients with patient risk factors, 55 % with an unknown source and 51 % with ≥ 3 blood culture bottles positive. Reasons for not performing echocardiography included patients who died prior to completion of investigation, patients receiving palliative care and therefore deemed not appropriate, or were not fit enough for ongoing investigations including TOE. Documentation of rationale for not conducting echocardiography was often lacking, although this may be due to lack of access to documentation.

Incidence of infective endocarditis.

10 patients were confirmed to have infective endocarditis, resulting in an incidence rate of 7.1 % (95 % CIs: 3.5–12.7 %) across all 140 patients with *E. faecalis* bacteraemia. The rate of IE increased to 13.0 % (6.4–22.6 %) for patients with monomicrobial *E. faecalis* bacteraemia (total 77 patients). 15.1 % of patients who underwent echocardiography were diagnosed with IE.

**Fig. 1:**
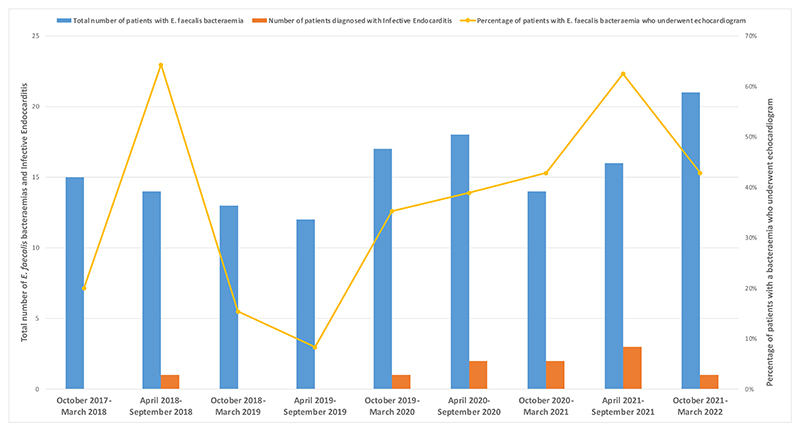
Number of patients with E. faecalis bacteraemia, confirmed endocarditis and echocardiography rate over time.

The incidence of endocarditis increased over time. The percentage of patients with *E. faecalis* bacteraemia who were diagnosed with endocarditis was 14.3 % in 2022, compared to 3.2 % in 2018, with a stepwise increase across consecutive years (Fig. 1).

Echocardiography rates of patients with *E. faecalis* bacteraemia were as follows: 2017––14 %, 2018––42 %, 2019––16 %, 2020––39 %, 2021––51 % and 2022–53 %, suggesting an overall increase in echocardiography use over time. Clearance cultures were obtained at the following rates: 2017––86 %, 2018––65 %, 2019––80 %, 2020––52 %, 2021––65 % and 2022––100 %.

All cases of endocarditis were diagnosed during the same admission as the bacteraemia. 7 patients without IE had recurrent bacteraemia. None of these patients were diagnosed with endocarditis on either episode, although 2 patients did not undergo echocardiography on the recurrent episode.

How do the risk characteristics of patients with endocarditis compare to those without endocarditis?

Factors which were associated with endocarditis were the presence of a patient risk factor for IE (as defined in methods), community acquired bacteraemia, monomicrobial bacteraemia and greater number of blood culture bottles positive (Table 1). Male patients were both more likely to acquire *E. faecalis* BSI and to develop endocarditis but this did not meet statistical significance. There was no age difference between the groups.

**Fig. 2:**
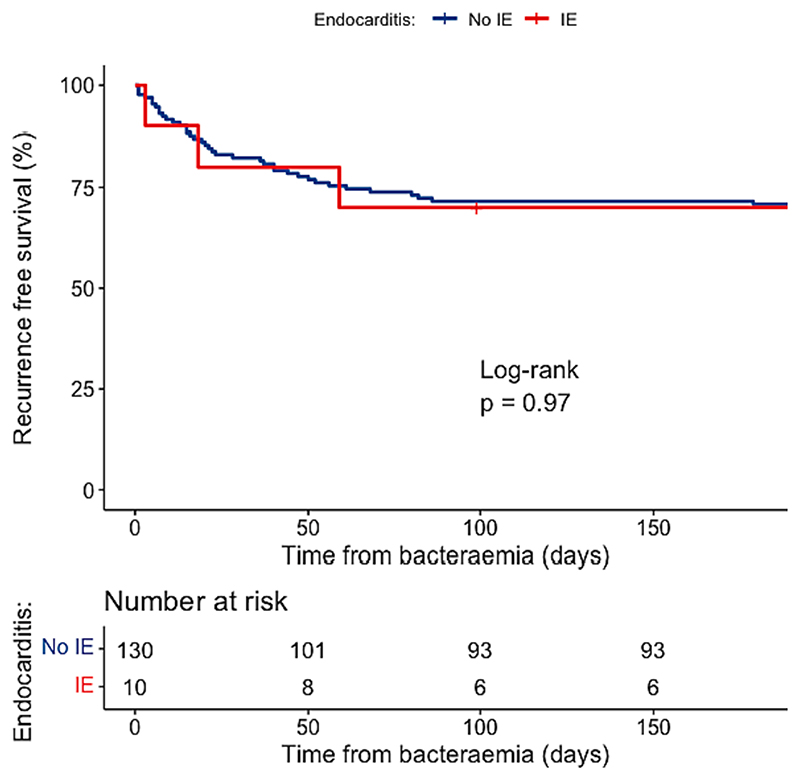
Recurrence free survival over 6 months from blood stream infection.

Diagnosis of IE was not associated with higher mortality either during admission or at 1 year. Recurrence free survival did not differ between those diagnosed with or without IE (Fig. 2).

## Discussion

Our study demonstrated an incidence of infective endocarditis in *Enterococcus faecalis* bacteraemia of 7 % (95 % CIs: 3.5–12.7 %) in all cultures and 13 % (6.4–22.6 %)

in monomicrobial cultures. Our findings are in keeping with literature prior to 2018, (Anderson, 2004; Pinholt, 2014; Malone, 1986; Bouza, 2015) but lower than the rates found by Østergaard and Dahl.

The current evidence regarding incidence rates of IE in *E. faecalis* bacteraemia is summarised in Table 2. Importantly, studies had differing inclusion criteria: varying between any *Enterococcus* species bacteraemia, *E. faecalis* only, and monomicrobial *E. faecalis* only, which makes comparison challenging.

Our study is most comparable in design to Østergaard’s study, which was also a retrospective review. However, Østergaard does not specify if all *E. faecalis* cultures were included or only monomicrobial cultures. This differentiation had a significant impact on our IE rates, and makes direct comparison difficult. It is important to note that Dahl did include polymicrobial cultures, and still found a significantly higher IE rate at 26 %.

When comparing patient demographics with those of Østergaard and Dahl, we found a male preponderance accounting for 69.3 % of *E. faecalis* bacteraemias, which was also shown in both Østergaard (72.5 %) and Dahl (74 %). However, the mean age of our cohort, at 66, was lower than Østergaard or Dahl, whose groups had mean ages of 73.7 and 74 respectively, and also younger than the average UK hospital adult inpatient (Hospital Admitted Patient Care Activity, 2021). This is likely due to the haematology and renal patients in our cohort, who had mean ages of 58 and 55 years respectively. Given that increasing age is associated IE risk, a younger population may partly explain our lower endocarditis rate.

A higher proportion of central venous access catheter (CVC) infections due to the over-represenation of haematology and renal patients in our cohort may also contribute to our lower incidence of IE. CVC related bacteraemias are generally of shorter duration due to the comparative ease of source control. 19.3 % of our bacteraemias were due to CVC infection, compared to a maximum of 11.6 % in Dahl (combined data CVC, wound, respiratory and dental infection). Østergaard did not report the source of bacteraemia.

The main reason for our lower incidence rate is likely to be under-use of echocardiography. Reasons for not performing echocardiography included patients who were palliative or not fit enough for ongoing investigation, under-appreciation of endocarditis risk, and access to echocardiography, with often long waiting lists for echocardiography.

Other factors which may play a role in our lower incidence rate include non-measurable factors affecting diagnosis, including differences in healthcare approach between the UK and Denmark, such as access to echocardiography or the threshold at which the clinician or patient may decide that further investigations are not in the patient’s best interests. Although there has been an increase of our rates of echocardiogram over the years, it is not clear if this was influenced by the publication of Østergaard and Dahl in 2019, or other factors such as the availability of echocardiograms.

The factors which increased risk in Dahl’s study were also corroborated by our findings, with community acquisition, patient risk factors, monomicrobial bacteraemia and longer duration of proven bacteraemia all being associated with higher IE likelihood. This is useful information for guiding investigations in future patients with *E. faecalis* bacteraemia.

It was also interesting that although 29 patients did not undergo echocardiography in Dahl *et al*, due to early discharge or patient decision, none developed clinical endocarditis subsequently. These patients may have been less comorbid with less complex infections given their prompt discharge, so would be likely to have a lower endocarditis risk. However, assuming this group had the same risk as the overall group, the probability of none developing endocarditis would be 0.016 %. This raises the question of whether performing routine echocardiography in a population which has not been stratified for risk could be associated with false positive diagnoses of endocarditis. However, given the significant morbidity and mortality associated with untreated endocarditis, over-diagnosis would be preferable to under-diagnosis.

Even when the above considerations are taken into account, it is probable that our diagnostic rates of endocarditis are too low when compared to other studies. Comparative under-use of echocardiography is likely to be the main factor in under-diagnosis.

The mortality rate of the patients was high, at 22.8 % during the admission, and 38 % within a year of the BSI. This was similar to the 40.7 % 1 year mortality found by Dahl *et al*. This reflects both the medical complexity of the patient cohort, as well as the significance of the bacteraemia itself.

Interestingly, we did not see a difference in mortality between patients with or without IE (albeit with a small sample size). In the context of likely under-diagnosis of IE this may suggest that missing cases may not be associated with worse outcomes. This may be because standard bacteraemia treatment may provide reasonable treatment of unidentified (and presumably milder) IE. Some patients may also have received extended antibiotic courses for other reasons (e.g. bone/joint infection) which could have treated IE despite a lack of formal diagnosis but this information was not available from records. Alternatively, it may be that mortality following *E. faecalis* bacteraemia is mainly due to underlying disease/comorbidity and not untreated infection.

The main limitations to our study are the relatively small numbers of patients, as although 140 patients were included, only 10 patients were diagnosed with endocarditis. Incomplete patient records were also a limitation.

As a result of this study, we have undertaken a number of interventions. Education sessions have been delivered to the Microbiology, Infectious Diseases and Cardiology departments, with a particular emphasis on the higher incidence of IE in *E. faecalis* bacteraemia, and on factors increasing the endocarditis risk to enable prioritisation of patients for echocardiography. We have also established a weekly endocarditis multi-disciplinary team meeting between Infectious Diseases, Microbiology and Cardiology. This provides opportunities for detailed discussion of individual patients, and improves collaborative working between specialties.

## Conclusions

Patients with *E. faecalis* bacteraemia are a complex group, often with multiple comorbidities and a significant mortality rate. It is increasingly recognised that *E. faecalis* is associated with a significant infective endocarditis risk, and that endocarditis incidence is increasing annually. Although the lower rate of IE in our cohort compared to recently published studies may be explained by a younger patient population and more CVC related infections, this is unlikely to be the full picture and may reflect previous, local diagnostic practice. Importantly, our current selection of patients for echocardiography does not correlate with the presence of risk factors for endocarditis. We have implemented a number of strategies to address this and will re-examine the data for the next 12 months to look for a change in diagnostic rates.

## Figures and Tables

**Table 1:** Proportion of patients with each characteristic in groups with and without endocarditis.

	Endocarditis within six months of BSI		
Variable	No, N = 130	Yes, N = 10	p-value
**Age Gender**	66 (55 – 79)	69 (61 – 75)	0.74 0.18
*Female*	42 (32)	1 (10)	
*Male*	88 (68)	9 (90)	
**Acquisition** *Community*	74 (57)	10 (100)	**0.006**
*Hospital*	56 (43)	0 (0)	
**Source of infection**			0.062
*Abdomen*	14 (11)	1 (10)	
*Line*	27 (21)	0 (0)	
*Other*	1 (0.8)	0 (0)	
*SSTI/bone/joint*	17 (13)	1 (10)	
*Unknown*	43 (33)	8 (80)	
*Urine*	28 (22)	0 (0)	
**Number of bottles positive**	2.00 (1.00 – 2.00)	4.00 (2.00 – 6.00)	**0.001**
***E. faecalis *mono-bacteraemia**	67 (52)	10 (100)	**0.002**
**Amoxicillin sensitivity**	128 (98)	10 (100)	> 0.99
**Any IE risk factor**	53 (41)	7 (70)	0.10
**IE risk factor**			**< 0.001**
*Abnormal native valve*	9 (7.0)	0 (0)	
*Cardiac device*	4 (3.1)	2 (20)	
*Immune suppression*	33 (26)	1 (10)	
*None*	78 (60)	3 (30)	
*Previous IE*	0 (0)	1 (10)	
*Prosthetic valve*	5 (3.9)	3 (30)	
*PWID*	1 (0.8)	0 (0)	
**Admission outcome**			0.29
*Death*	28 (22)	4 (40)	
*Discharge*	102 (78)	6 (60)	
**One-year mortality**	49 (38)	4 (40)	> 0.99
Median (IQR); n (%)			
Wilcoxon rank sum test; Fisher’s exact test			

**Table 2:** Summary of previous findings of infective endocarditis rates in *E. faecalis* bacteraemia.

Study	Number of patients	Included	Rate of endocarditis
Malone *et al* 1986 (Malone, 1986)	55	Bacteraemia with any enterococcal species	10 %
Anderson *et al* 2004 (Anderson, 2004)	1255	Bacteraemia with any enterococcal species	3.3 %
Pinholt *et al* 2014 (Pinholt, 2014)	700	Monomicrobial *E.faecalis* bacteraemia	13.3 %
Bouza *et al.* 2015 (Bouza, 2015)	1515	*E.faecalis* bacteraemia – mono-microbial and mixed	4.29 %
Dahl *et al* 2019 (Dahl, 2019)	344	*E.faecalis* bacteraemia – mono-microbial and mixed – all underwent proactive echo	26 %
Østergaard *et al* 2019 (Østergaard, 2019)	4700	*E.faecalis* bacteraemia – unclear if mono-microbial only or mixed included	16.7 %
Berge *et al* 2019 (Berge, 2019)	397	Only mono-microbial *E. faecalis* bacteraemia	11.1 %
Fernandez-Hidalgo *et al* 2019 (Fernandez-Hidalgo, 2019)	79	*E.faecalis* bacteraemia – monomicrobial and mixed	14 %
